# The evolution of socioeconomic status-related inequalities in maternal health care utilization: evidence from Zimbabwe, 1994–2011

**DOI:** 10.1186/s41256-016-0021-8

**Published:** 2017-01-05

**Authors:** Marshall Makate, Clifton Makate

**Affiliations:** 1 Department of Economics, State University of New York at Albany, Albany, NY 12222 USA; 2 UNEP Tongji Institute of Environment for Sustainable Development, Tongji University, Shanghai, China

**Keywords:** Socioeconomic status-related inequality, Maternal health care utilization, Erreygers concentration index, Zimbabwe

## Abstract

**Background:**

Inequalities in maternal health care are pervasive in the developing world, a fact that has led to questions about the extent of these disparities across socioeconomic groups. Despite a growing literature on maternal health across Sub-Saharan African countries, relatively little is known about the evolution of these inequalities over time for specific countries. This study sought to quantify and explain the observed differences in prenatal care use and professional delivery assistance in Zimbabwe.

**Methods:**

The empirical analysis uses four rounds of the nationwide Zimbabwe Demographic and Health Survey administered in 1994, 1999, 2005/06 and 2010/11. Two binary indicators were used as measures of maternal health care utilization; (1) the receipt of four or more antenatal care visits and (2) receiving professional delivery assistance for the most recent pregnancy. We measure inequalities in maternal health care use using the Erreygers corrected concentration index. A decomposition analysis was conducted to determine the underlying drivers of the measured disparities.

**Results:**

The computed concentration indices for professional delivery assistance and prenatal care reveal a mostly pro-rich distribution of inequalities between 1994 and 2011. Particularly, the concentration index [95% confidence interval] for the receipt of prenatal care was 0.111 [0.056, 0.171] in 2005/06 and 0.094 [0.057, 0.138] in 2010/11. For professional delivery assistance, the concentration index stood at 0.286 [0.244, 0.329] in 2005/06 and 0.324 [0.283, 0.366] in 2010/11. The pro-rich inequality was also increasing in both rural and urban areas over time. The decomposition exercise revealed that wealth, education, religion and information access were the underlying drivers of the observed inequalities in maternal health care.

**Conclusions:**

In Zimbabwe, socioeconomic disparities in maternal health care use are mostly pro-rich and have widened over time regardless of the location of residence. Overall, we established that inequalities in wealth and education are amongst the top drivers of the observed disparities in maternal health care. These findings suggest that addressing inequalities in maternal health care utilization requires coordinated public policies targeting the more poor and vulnerable segments of the population in Zimbabwe.

## Background

Across the world, studies have shown that disparities in health do exist, mostly favor the high-income groups and are more pronounced in some countries than others [1–5]. However, some of these studies have mainly focused on measuring and explaining inequalities in health in the developed world with few studies for developing countries starting to emerge. Regardless of the setting, there is general agreement in the empirical literature that individuals from higher socioeconomic status groups enjoy better health compared to their counterparts from lower socioeconomic status groups [6, 7]. Achieving equity in maternal health care is one of the most stressed and notable public health policy concern shared in almost every country in the world and requires that individuals with the same maternal health care needs be granted the same opportunities to access health care [8]. In Zimbabwe, for example, despite efforts to improve access to maternal health care utilization over the years, inequality in maternal health care remains a public health concern [9]. To date, the government of Zimbabwe has implemented many policies to improve access to maternal health care including the Primary Health Care (PHC) of the mid-1980s and the Maternal and Neonatal Health (MNH) roadmap 2007–2015 launched in 2009 among others [10]. It is also important to note that Zimbabwe has witnessed one of the worst economic crisis in its history that saw the deterioration in the major sectors of the economy including health, manufacturing, and farming [11, 12]. The degradation in the quality of health as a result of the exodus of qualified health professionals to neighboring countries and abroad has contributed to inequalities in health [12]. The increase in user fees in health in 1993–94 is plausibly responsible for the widening gap between the poor and rich in the country. Thus, it is imperative for emerging research to focus on the extent to which access to maternal health care is equitable among the individuals in need than an emphasis on the determinants of access to these services.

Previous studies examining equities in health service use in high-income countries especially in the Organization for Economic Cooperation and Development (OECD) region and the U.S. have established a more pro-rich concentration of health care utilization [1, 13, 14]. Related studies conducted in Asia have also confirmed a pro-rich distribution of health care use among the most affluent segments of the population [15]. In other countries such as Nepal, a significant pro-rich pattern of inequalities in health care use has been found [16]. Other studies have also found a pro-rich distribution in disparities in maternal health care use [4, 17–19]. However, it is imperative to note that there have been numerous studies in various countries documenting the causes of inequalities in maternal health care use and child mortality [4, 19–23], while surprisingly little is known in the context of Zimbabwe.

This study seeks to fill this gap by focusing on Zimbabwe – an important and yet understudied case in the literature. Specifically, we measure and explain wealth-related inequalities in prenatal care use, and professional delivery assistance using the G Erreygers [24] corrected concentration index. We document the evolution over time since 1994 and provide a decomposition to determine the underlying factors explaining the observed inequalities in maternal health care in 2005/06 and 2010/11 following the guidelines laid out in O O’Donnell, E van Doorslaer, A Wagstaff and M Lindelow [21].

## Methods

### Measuring inequalities in maternal health care utilization

Our primary measure of socioeconomic status-related inequalities in maternal health care utilization is by means of the widely employed concentration index [25]. Derived from the concentration curve, the concentration index measures the extent to which a health care outcome is associated with inequality in a measure of socioeconomic status, typically income [26]. Since the purpose of this study is on measuring and explaining wealth-related inequalities in maternal health care utilization, defined mainly by binary variables, we employ the corrected version of the concentration index which is suitable for bounded variables as suggested by G Erreygers [24]. One of the drawbacks often mentioned about the standard concentration index is with regards to its overdependence on the mean of the health variable. This limitation is particularly problematic if one is interested in comparing populations with different average health levels [24]. Besides, in the case of a binary variable, the standard concentration index may not always be restricted to the [−1, + 1] interval [27]. Moreover, the standard concentration index has also been shown to violate the “mirror property,” an assumption that says that inequalities in health should “mirror” variations in ill-health [28]. For the noted reasons, we use the G Erreygers [24] corrected concentration index which is algebraically expressed as follows:

1 $$ E(h)=8cov\left({h}_i,{R}_i\right) $$

where $E(h)$ is the Erreygers corrected concentration index, ${h}_i$ is the maternal health outcome of interest, ${R}_i$ is the individual or respondent’s relative rank in the household wealth distribution, The size and magnitude of $E(h)$ reflects the strength and variability in the maternal health outcome of interest [21]. Positive (negative) values of $E(h)$ indicate a pro-rich (pro-poor) distribution. To deduce more meaningful inferences A Wagstaff, E van Doorslaer and N Watanabe [29] suggested a way of decomposing the measured inequalities in health into their specific determining components using the following linear equation:

2 $$ {h}_i={\beta}_0+\underset{k=1}{\overset{K}{\varSigma }}{\beta}_k{x}_{ik}+\underset{l=1}{\overset{L}{\varSigma }}{\beta}_l{z}_{il}+{\varepsilon}_i $$

where ${h}_i$ is the health measure, ${x}_{ik}$, and ${z}_{il}$ are the need and non-need related characteristics. Equation (2) is estimated using an ordinary least square (OLS) regression model [1].

### Data source

Our empirical analysis utilizes data from four rounds of the nationally representative Demographic and Health Survey for Zimbabwe (henceforth ZDHS) conducted in 1994, 1999, 2005/2006, and 2010/2011. The survey is part of the global MEASURE DHS program currently carried out in more than 40 developing countries. This data is made available after a formal request at (http://dhsprogram.com/data/available-datasets.cfm). The ZDHS gathers detailed health information for women of reproductive ages 15–49 and their children. The Zimbabwe National Statistics Agency (ZIMSTAT) conducted all the four rounds of the survey with collaborative assistance from numerous national and international organizations.

The survey used a stratified two-stage cluster sample design based on the Zimbabwe population census of 1992 and 2002. The 1994 and 1999 ZDHS utilized the 1992 population census while the 2005/06 and 2010/11 ZDHS utilized the 2002 population census sampling frames. The first stage involved a random sampling of the enumeration areas followed by a random sampling of households (excluding families from institutional facilities such as army barracks, hospitals, police camps, and boarding schools) at the second stage. This dataset is fitting for our analysis since it contains detailed information on the household structure, asset ownership, health, and labor market participation including education characteristics for all the family members. An excellent guide to the DHS data is also available in SO Rutstein and G Rojas [30].

The inquiry in this study uses the individual recode component of the ZDHS which contains detailed health information of the interviewed women. The ZDHS records information on maternal health care utilization of the most recent pregnancy that occurred in the five years before each survey. Thus, we restrict our analysis to the last birth that took place five years before each survey for each interviewed woman. From the original sample of 21,601 observations from the pooled ZDHS 1994, 1999, 2005/2006 and 2010/2011 data, we are left with 13,506 women with non-missing observations on our outcome variables. All the estimates are weighted to be nationally representative. The initial survey weights are adjusted to account for the possible effect of pooling across surveys. Specifically, we re-scale each survey’s total weight to sum to one thus manually preserving the original probability of sampling within each survey. Here we make the assumption that the overall population did not significantly change over the analysis period to the extent of altering our conclusions.

### Outcome variables

This study uses two measures of maternal health care utilization derived from the various questions asked during the ZDHS. First, we consider the receipt of four or more antenatal care visits as our measure for prenatal care use. Prenatal care is the medical attention given to women during (excluding delivery period) pregnancy [31]. As recommended by the World Health Organization, women in developing countries with less complicated pregnancies are encouraged to complete at least four antenatal care visits during the course of the pregnancy [31]. We measure antenatal care as a binary variable taking 1 if the woman completed four or more prenatal care visits during pregnancy and 0 otherwise. Second, we measure professional delivery assistance using a binary indicator taking 1 if the woman received delivery assistance by a medical doctor, auxiliary nurse, midwife or other trained health professional and 0 otherwise.

### Explanatory variables

The prospect of completing four or more prenatal care visits and of seeking professional delivery assistance is thought to depend on a set number of characteristics including individual demographic, household, and locational factors. The choice of these variables is primarily guided by the current empirical literature on maternal health care utilization in developing and developed countries. These variables include binary indicators for the age of the woman at time of birth (13–19; 20–24; 25–29; 30–34; 35–39; 40–44; and 45–49), education level (no education; completed primary; secondary or higher), contraceptive usage (yes = 1), marital status (separated; never married; married), employment status (employed = 1), religious beliefs (Christian; apostolic church member; other religion), access to information (watch television, listen to the radio and read newspapers), previously terminated pregnancy (yes = 1). We also included dummy indicators for the household wealth (poorest; poorer; average; rich; richer). To control for geographical differences, we included dummy indicators for urban/rural status (urban = 1) and provinces (Manicaland; Mashonaland Central; Mashonaland East; Mashonaland West; Matabeleland North; Matabeleland South; Midlands; Masvingo; Harare; Bulawayo).

### Measuring socioeconomic status using the asset index

This study makes use of an asset-based household wealth index as a measure of socioeconomic status, created using Principal Components Analysis (PCA) [32]. Numerous other studies have utilized the asset index as a measure of socioeconomic status in explaining inequalities in various health outcomes [21, 33, 34]. The ZDHS creates this index using information on household ownership of personal assets and home dwelling characteristics. A more comprehensive description of how this index is computed can be found in SO Rutstein and K Johnson [35].

## Results

### Descriptive statistics

Table 1 presents the survey-weighted means and standard deviations of all the variables used in the analysis stratified by the year of survey. Our sample is predominantly Christian (54.3%) and mostly living in rural areas (68.8%). The average education of the respondents appears to have improved over time with 67.2% of respondents in 2010/11 having completed secondary school or higher compared to only 37.4% in 1994. The share of women in gainful employment has declined over time from 52% in 1994 to 36% in 2010/11. The overall marital status distribution indicates that nearly 84.1% of the women in our sample were married as observed at the time of the survey. Overall, approximately 61.1% of the women practiced family planning (i.e., indicated using a modern family planning method), 39% read newspapers at least once a week, 51.1% listened to the radio at least once a week, and nearly 10.5% reported having terminated a pregnancy in the past. The share of women living in urban areas stood at about 31.2% and ranged from 26.7% in 1994 to about 31.2% in 2010/11. The average proportion of interviewed women in each province appears to be stable over time with Bulawayo having an overall lowest share of about 5.2% and Harare having the overall largest (15.1%).

**Table 1:** Summary statistics of variables used in the analysis

	Overall		1994		1999		2005/06		2010/11	
Variables	Mean	SD	Mean	SD	Mean	SD	Mean	SD	Mean	SD
Age 13–19	0.154	0.361	0.147	0.354	0.166	0.372	0.156	0.363	0.145	0.352
Age 20–24	0.324	0.468	0.319	0.466	0.327	0.469	0.339	0.474	0.309	0.462
Age 25–29	0.235	0.424	0.211	0.408	0.231	0.421	0.232	0.422	0.259	0.438
Age 30–34	0.160	0.367	0.175	0.380	0.138	0.344	0.160	0.366	0.174	0.379
Age 35–39	0.087	0.283	0.100	0.300	0.098	0.297	0.073	0.260	0.082	0.275
Age 40–44	0.032	0.177	0.040	0.195	0.034	0.181	0.033	0.178	0.025	0.156
Age 45–49	0.007	0.082	0.008	0.087	0.007	0.084	0.007	0.081	0.006	0.078
Marital status – married	0.841	0.366	0.864	0.343	0.838	0.368	0.814	0.389	0.851	0.356
Employed	0.438	0.496	0.520	0.500	0.526	0.499	0.364	0.481	0.360	0.480
No education	0.058	0.234	0.126	0.332	0.065	0.247	0.041	0.197	0.017	0.131
Primary education	0.393	0.489	0.500	0.500	0.436	0.496	0.352	0.478	0.311	0.463
Secondary education	0.548	0.498	0.374	0.484	0.499	0.500	0.607	0.488	0.672	0.470
Religion – Christian	0.543	0.498	0.495	0.500	0.815	0.388	0.435	0.496	0.408	0.492
Reads newspapers at least one a week	0.390	0.488	0.435	0.496	0.390	0.488	0.388	0.487	0.359	0.480
Listens to the radio at least once a week	0.511	0.500	0.383	0.486	0.580	0.494	0.528	0.499	0.522	0.500
Family planning	0.611	0.488	0.575	0.494	0.596	0.491	0.643	0.479	0.621	0.485
Terminated pregnancy	0.105	0.306	0.120	0.325	0.107	0.310	0.097	0.296	0.098	0.297
Wealth quintile 1 – poorest	0.219	0.413	0.241	0.428	0.195	0.397	0.228	0.420	0.216	0.412
Wealth quintile 2	0.192	0.394	0.183	0.386	0.179	0.383	0.201	0.401	0.205	0.404
Wealth quintile 3	0.183	0.387	0.179	0.384	0.186	0.389	0.174	0.379	0.191	0.393
Wealth quintile 4	0.222	0.415	0.211	0.408	0.233	0.423	0.220	0.414	0.219	0.414
Wealth quintile 5 – (richest)	0.184	0.388	0.186	0.389	0.207	0.405	0.177	0.382	0.168	0.374
Urban resident	0.312	0.464	0.267	0.442	0.346	0.476	0.313	0.464	0.312	0.463
Manicaland province	0.137	0.344	0.131	0.337	0.151	0.358	0.121	0.326	0.142	0.349
Mashonaland central province	0.101	0.301	0.087	0.282	0.094	0.293	0.111	0.315	0.106	0.308
Mashonaland east province	0.090	0.286	0.102	0.302	0.087	0.282	0.078	0.268	0.096	0.295
Mashonaland west province	0.110	0.313	0.116	0.320	0.099	0.299	0.101	0.301	0.125	0.330
Matabeleland north province	0.060	0.237	0.077	0.266	0.054	0.226	0.064	0.245	0.049	0.215
Matabeleland south province	0.052	0.223	0.058	0.234	0.059	0.236	0.045	0.207	0.048	0.214
Midlands province	0.131	0.338	0.137	0.343	0.123	0.329	0.143	0.350	0.124	0.329
Masvingo province	0.117	0.322	0.102	0.303	0.103	0.304	0.149	0.356	0.112	0.316
Harare province	0.151	0.358	0.140	0.347	0.168	0.373	0.138	0.345	0.156	0.363
Bulawayo province	0.052	0.221	0.051	0.221	0.061	0.240	0.051	0.219	0.043	0.202
Observations	13506		2218		2818		4073		4397	

Notes: All estimates are weighted to be nationally representative. Due to rounding, other statistics might not sum to one

**Fig. 1:**
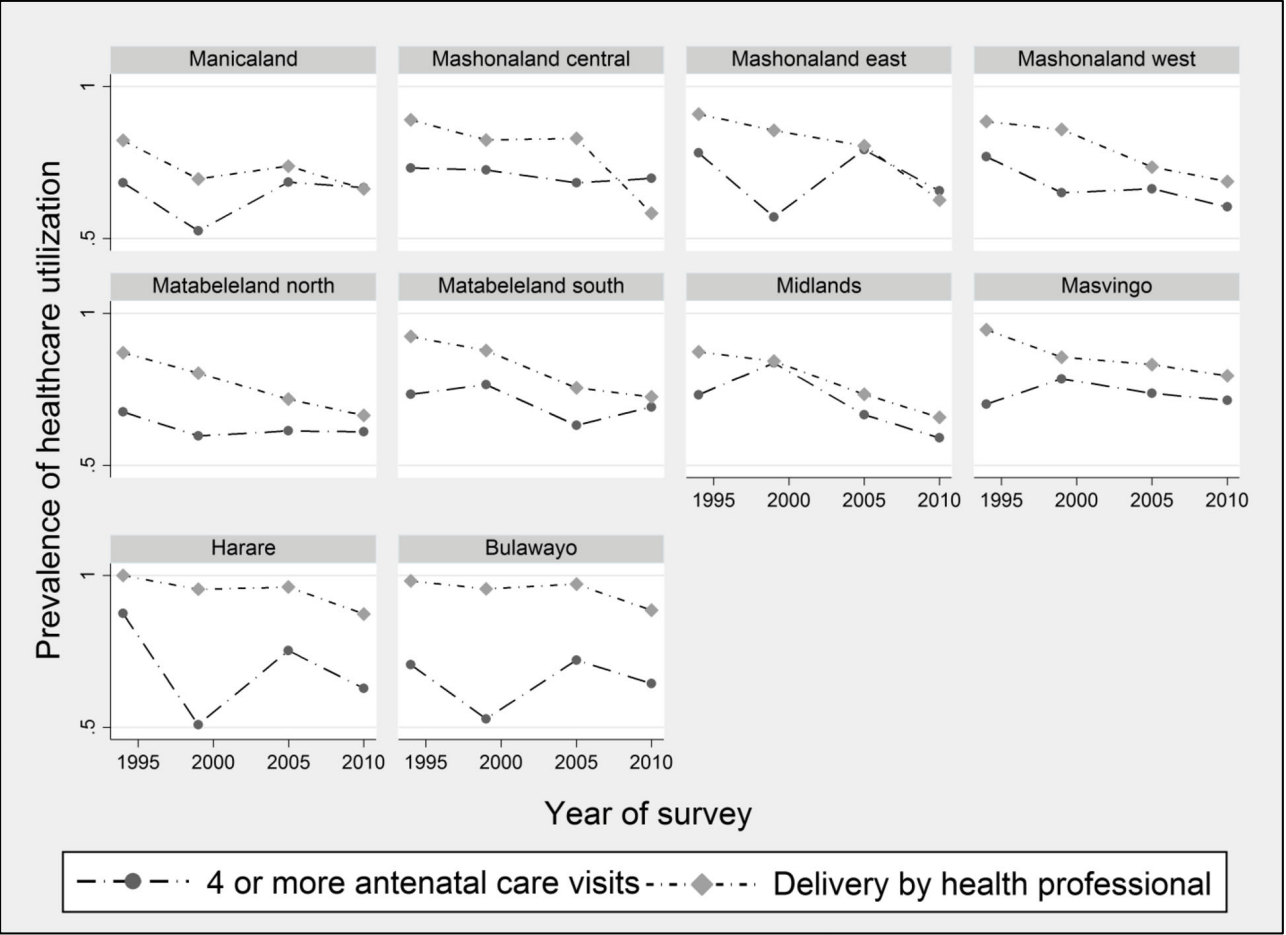
Prevalence of maternal health care utilization by region of residence in Zimbabwe, 1994–2011

Figure 1 presents the trends in maternal health care utilization in Zimbabwe’s ten provinces. While the prevalence of health care utilization appears to vary across regions, we observe nearly similar patterns in some of the provinces. For instance, the trends in professional delivery assistance and prenatal care seem to be somewhat similar in Manicaland, Mashonaland Central, East, and West, Matabeleland North, Harare, and Bulawayo provinces. The prevalence in prenatal care use in Matabeleland South, Midlands, and Masvingo appear to be somewhat different from the observed patterns in other regions. Specifically, we observe an initial rise in prenatal care prevalence in the 1994–1999 period followed by a persistent and declining trend over the period 2000–2010. Overall, the prevalence rates for maternal health care use in the 2010/11 period appear to have worsened compared to their 1994 levels in nearly all the provinces.

**Fig. 2:**
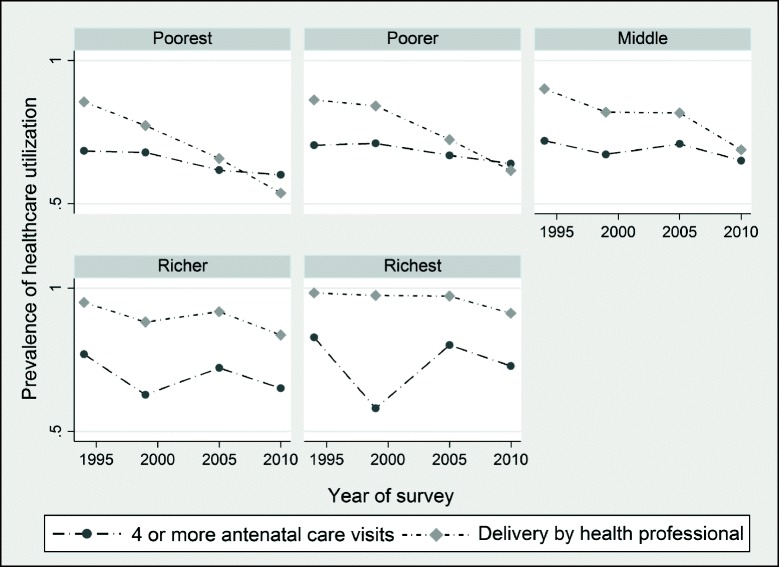
Prevalence of maternal health care utilization by household wealth group in Zimbabwe, 1994–2011

Figure 2 shows the trends in maternal health care utilization by household wealth quintile. According to Fig. 2, the prevalence rates for women in the bottom three wealth quintiles (poorest, poorer, and average) appear somewhat lower than those in the top two wealth quintiles (richer and richest). Also, we observe a rather steeper and declining trend in maternal health care use for individuals in the bottom three wealth groups. Women from wealthier families (richer and richest) appear to have had high utilization rates over time. However, the prevalence of prenatal care use for women in the top wealth quintiles seems to show an unstable pattern over time compared to those in the bottom three wealth quintiles.

**Fig. 3:**
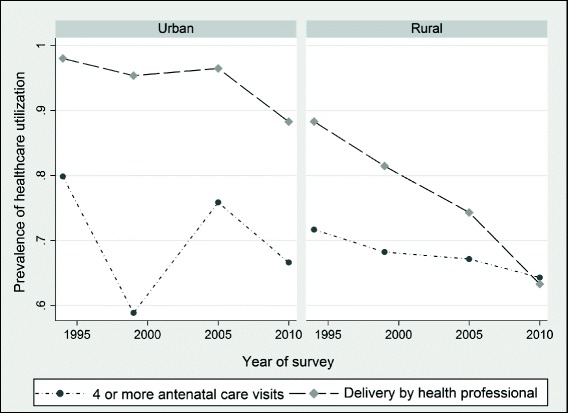
Prevalence of maternal health care utilization by rural or urban status in Zimbabwe, 1994–2011

Figure 3 depicts the prevalence rates for women living in urban and rural areas. As expected, women living in urban communities appear to have had better access to professional delivery assistance compared to their rural counterparts. Regarding prenatal care, urban women seem to have maintained a very unstable pattern in utilization compared to their rural counterparts who have experienced a steady decline in use over time. Overall, we observe lower utilization rates for both rural and urban communities in 2010/11 compared to 1994 for all the maternal health care outcomes.

### Trends in inequalities in maternal health care use

**Fig. 4:**
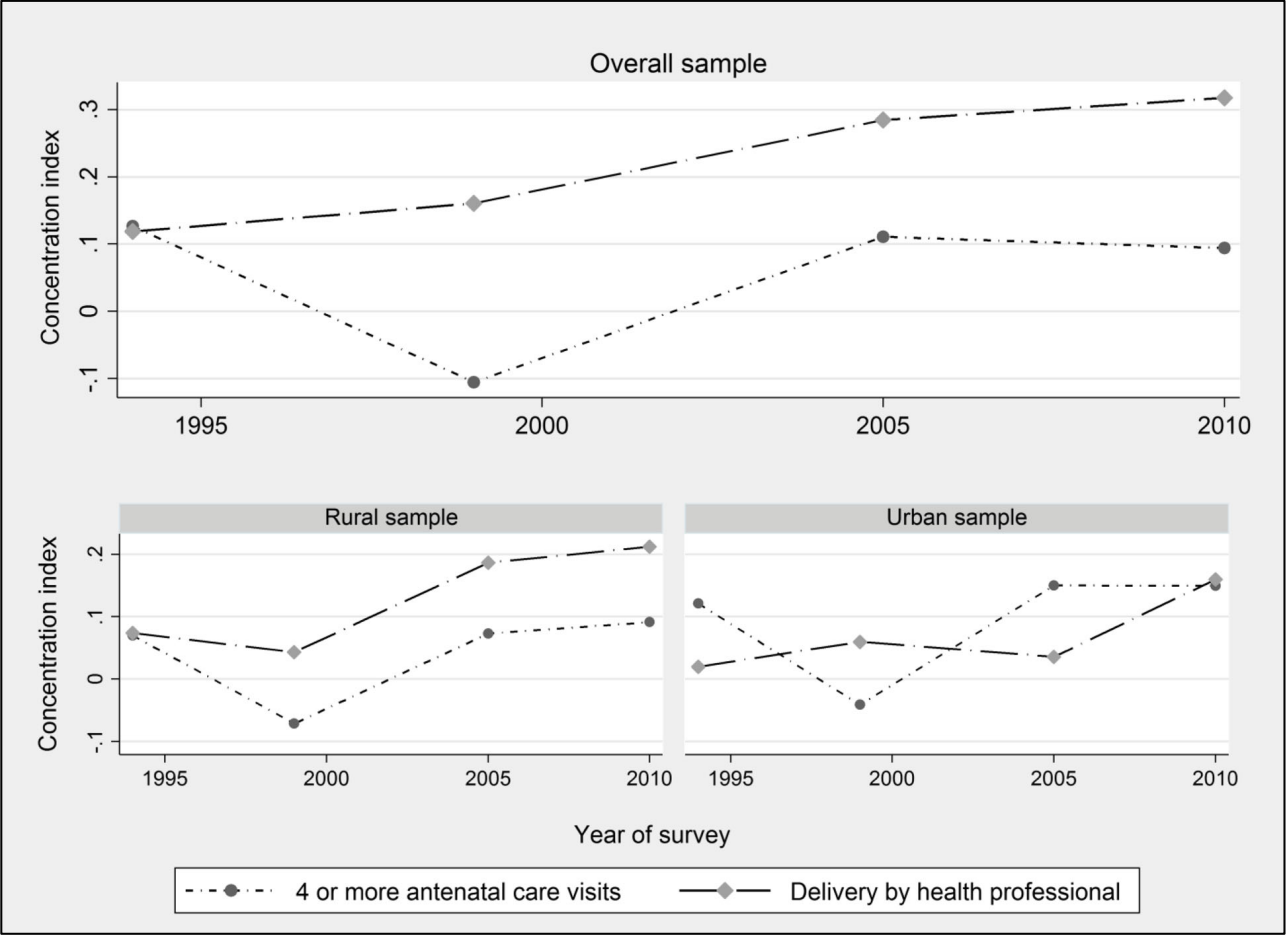
The evolution of inequalities in maternal health care in Zimbabwe, 1994–2011

Figure 4 shows a graphical presentation of the corrected concentration indices for prenatal care, and professional delivery assistance for the overall, rural and urban samples. The concentration indices are calculated using O O’Donnell, S O’Neill, T Van Ourti and B Walsh [36] *conindex* command and are weighted to be nationally representative including clustering at the primary sampling unit to appropriately adjust the standard errors. The top panel of Fig. 4 displays the overall distribution of inequalities in maternal health care since 1994. The overall trends in disparities in prenatal care use show a pro-rich distribution in 1994, 2005/06 and 2010/11 with a pro-poor distribution observed in 1999. Inequalities in professional delivery support have also been pro-rich over the period under study. Specifically, it is evident from Fig. 4 that inequalities in professional delivery assistance have worsened over time and have mostly been pro-rich.

The bottom panel of Fig. 4 shows the distribution of inequalities for rural and urban samples. Regardless of the location of residence (rural or urban), the distribution of professional delivery support has mostly been pro-rich. For the rural sample, inequalities in professional delivery assistance initially declined over the 1994–1999 period and after that show a rising trend. A similar pattern is also noticeable in the distribution of disparities in the receipt of four or more antenatal care visits. For the rural sample, inequalities in prenatal care use were mostly pro-rich in 1994 while the 1999 survey data reveals a rather pro-poor distribution. The period from 2000 and beyond suggests that differences in prenatal care utilization for the rural sample have been pro-rich. The pattern of inequalities over time also reveals a widening gap between the rural wealth-poor and the rural wealth-rich individuals. For the urban sample, similar conclusions can also be drawn. Notably, we can conclude from Fig. 4 that inequalities in professional delivery assistance have mostly been pro-rich since 1994. Concerning prenatal care utilization, we observe a shift from a pro-rich to pro-poor distribution between 1994 and 1999 and a pro-rich distribution thereafter.

### Decomposition of socioeconomic status-related inequalities in maternal health care

To better understand the reasons why disparities in maternal health care have widened over time and to the advantage of the wealth-rich, we conducted a decomposition of the measured disparities in prenatal care, and professional delivery assistance. This exercise allows us to measure the contribution of each explanatory variable to the measured inequalities in maternal health care. For brevity, we only present the decomposition results using the 2005/06 and 2010/11 survey data. The coefficient estimates from the OLS models estimated using equation (2) are also not shown here (results available upon request).

Table 2 shows the absolute and percent contributions of each explanatory variable to the overall inequalities in maternal health care use. The concentration indices for prenatal care and professional delivery assistance including their 95% confidence intervals (in brackets) are shown in the bottom section of Table 2. The estimates indicate that the concentration index for prenatal care was 0.111 [0.056, 0.171] in 2005/06 and 0.094 [0.057, 0.138] in 2010/11, suggesting a pro-rich distribution in inequalities. The estimates for professional delivery assistance reveal a concentration index of 0.286 [0.244, 0.329] in 2005/06 and 0.324 [0.283, 0.366] in 2010/11. All the indices are significant at the 1% significance level.

**Table 2:** Contributions of regressors in percent (%) of concentration index

	Four or more prenatal care visits				Professional delivery assistance			
	2005/06		2010/11		2005/06		2010/11	
Variables	Contribution	%	Contribution	%	Contribution	%	Contribution	%
Household wealth	0.0175	45.84	0.0258	71.79	0.0170	36.14	0.0406	64.23
Age	0.0012	3.47	−0.0016	−4.61	0.0020	4.40	0.0018	2.99
Employed	0.0007	1.87	0.0005	1.47	0.0003	0.65	0.0004	0.57
Education	0.0103	26.69	0.0066	18.20	0.0041	8.80	0.0039	6.19
Religion	0.0046	12.01	0.0081	22.69	0.0029	5.98	0.0042	6.66
Marital status	−0.0084	−21.93	−0.0048	−13.34	0.0020	4.11	0.0038	5.94
Read newspapers	0.0021	5.42	0.0093	26.04	0.0038	8.12	0.0044	6.92
Listen to radio	0.0092	23.95	0.0055	15.23	0.0015	3.12	0.0002	0.37
Health insurance	0.0049	12.88	0.0044	12.13	0.0016	3.48	0.0019	2.96
Family planning	0.0002	0.58	−0.0011	−3.03	−0.0001	−0.23	0.0003	0.52
Terminated pregnancy	−0.0006	−1.48	0.0001	0.27	−0.0004	−0.90	−0.0001	−0.12
Urban residence	0.0082	21.35	−0.0014	−3.99	0.0141	30.18	0.0053	8.36
Region (nine provinces)	−0.0197	−51.81	−0.0250	−69.64	−0.0035	−7.27	−0.0054	−8.56
Residual		21.15		26.76		3.42		2.98
Total	0.0302	78.85	0.0264	73.24	0.0453	96.58	0.0613	97.02
Erreygers corrected concentration index	0.111***[0.056, 0.171]		0.094***[0.057, 0.138]		0.286***[0.244, 0.329]		0.324***[0.283, 0.366]	

Notes: ***Statistical significance at the 1% level. Estimates are weighted to be nationally representative. Contribution = the absolute contributions of explanatory variables to the concentration index. The corrected concentration indices together with their 95% confidence intervals (in brackets) are shown at the bottom of the table

The results for the decomposition indicate that household wealth explains a large share of the observed inequalities in maternal health care utilization between 2005/06 and 2010/11. Specifically, household wealth explains approximately 45.84 and 71.79% of the observed inequalities in prenatal care utilization in 2005/06 and 2010/11, respectively. Concerning professional delivery assistance, household wealth accounts for nearly 36.14% in 2005/06 and 64.23% in 2010/11. The positive sign on household wealth’s contribution implies that if household wealth was distributed equally across the population, then, the observed inequalities in maternal health care would be lower by the corresponding percentages as noted earlier.

Education is another important factor accounting for a sizeable share of the observed inequalities in maternal health care. The results show that if the distribution of education was uniformly distributed across the population of pregnant women, inequalities in prenatal care use would have been 26.69 and 18.20% lower in 2005/06 and 2010/11, respectively. However, education only explains about 8.8 and 6.19% of the inequalities in professional delivery assistance observed in 2005/06 and 2010/11, respectively. Information access through reading newspapers and magazines as well as listening to the radio also plays an important role in explaining the observed inequalities in maternal health care. We find that nearly 23.95 and 15.23% of the observed inequalities in prenatal care in 2005/06 and 2010/11 respectively can be explained by information acquisition through listening to the radio. The contribution of radio listenership to inequalities in professional delivery assistance appears to be somewhat low (below 5%) in 2010/11 while reading newspapers account for nearly 5.42 and 26.04% of the observed inequalities in prenatal care use in 2005/06 and 2010/11, respectively. The contribution of reading newspapers on inequalities in professional delivery assistance was below 10% over the two years. The findings in Table 2 also show that health insurance coverage accounts for a negligible and statistically insignificant share of the observed disparities in prenatal care and professional delivery assistance in 2005/06 and 2010/11.

## Discussion

In this paper, we have measured wealth-related inequalities in the receipt of four or more antenatal care visits, and professional delivery assistance using the corrected concentration index as suggested by G Erreygers [24]. An effort was also made to identify the underlying factors explaining the observed inequalities in maternal health care use over time. To the best of our comprehension, this is the first study for Zimbabwe that makes an attempt to document the evolution of inequalities in maternal health care and consequently explain the underlying drivers over the years. We found a pro-rich distribution in inequalities in professional delivery assistance over the 1994-2010/11 periods. The concentration indices for prenatal care use reveal a pro-rich distribution of disparities in 1994, 2005/06 and 2010/11 with a pro-poor distribution observed in 1999. The decomposition analysis of the wealth-related inequalities in maternal health care use demonstrated that household wealth was amongst the most important factors explaining the observed differences in maternal health care in Zimbabwe. Overall, these results corroborate the findings in previous studies [4, 17, 19].

The results indicate that inequality in household wealth was found to be one of the most influential contributors of the observed differences in prenatal care and professional delivery assistance in 2005–2011. This result makes intuitive sense given the documented rise in poverty levels in the country since the mid-1990s [37]. Also, the hyperinflationary environment that prevailed during the 2000–2008 crisis period worsened the plight of ordinary Zimbabweans primarily those residing in the rural areas (nearly 60% of Zimbabwe’s population lives in the countryside). The fact that the overall contribution of wealth has significantly increased between 2005/06 and 2010/11 suggests a further deterioration in the living standards among Zimbabweans and hence explaining the observed inequalities in maternal health care use. These findings here corroborate the conclusions made in previous related studies for developing countries that demonstrate the existence of socioeconomic status-related (socioeconomic status as measured by household wealth) inequalities in maternal care services in the dimensions of wealth [3, 4, 17, 19].

Apart from the fact that poverty levels have increased in Zimbabwe over the years [37], the rise in user fees associated with access to maternal health care services as noted in N Matshalaga [38], is also a contributory factor towards the marked increase in inequalities in maternal health care. As noted in previous studies, affordability of maternal health care services plays an essential role in shaping the overall demand of such services [39–41]. The devastating impact of the economic crises experienced since 2000 has largely contributed to the health personnel exodus and deterioration in health infrastructure in many parts of Zimbabwe, thus, resulting in limited access to the essential maternal health care services needed by pregnant women [12]. As the crisis led to the massive impoverishment of households particularly those in rural areas, the relatively better-off had better access to maternal health care.

The analysis in this study also singled out education as amongst the important predictors of inequalities in maternal health care. Education is widely regarded as an important predictor of maternal health care services. Particularly, previous studies have labeled schooling as a source of exclusion to the use of maternal health care services [42–44]. As noted in M Grossman [45] and many other studies, education has been singled out to be a significant correlate of good health and use of health care services [45–47]. Recent studies in sub-Saharan Africa have linked maternal education to improvements in child survival including increased maternal health care utilization and other healthy behaviors [48, 49]. In Zimbabwe, while schooling appears to explain a fair share of the observed inequalities in maternal health care use, its contribution has declined over time. The marked decline in the contribution of education might partly be attributed to the successes of the public school system [50]. Moreover, among African countries, Zimbabwe ranks highly regarding general literacy rates [51], an important and well-known determinant of population health [52].

The analysis in this study also found that religion contributes a fair proportion of the measured disparities in maternal health care. Most importantly, the study established that the overall contribution of religion to the observed inequalities in maternal health care has increased from 12.01% in 2005/06 to 22.69% in 2010/11. The marked increase in religion’s contribution highlights the importance of religion in explaining the measured inequalities in maternal care. The link between religion and use of maternal care services is well established [42–44, 53]. Over the last few years, Zimbabwe has witnessed an increasing number of apostolic section churches (Mapostori) and consequently their followers. Ultra-conservative apostolic members are believed to shun the use of modern medicine while believing in spiritual healing even in instances of severe sickness, pregnancy or other health-related matters [54, 55]. A previous study for Zimbabwe found that women affiliated with this particular church are 25% less liable to utilize the same health services than those from other religious sections [44]. Given that Zimbabwe’s rural and many parts of urban areas are home to these churches and mostly attended by poor people, it is more likely that religious beliefs have largely contributed to the observed widening gap in inequalities in maternal health care use.

The observed pro-rich distribution of disparities in maternal health care can also be attributed to many other factors including the decline in health infrastructure attributed to the economic crisis experienced in the 2000–2008 period. The combination of health personnel exodus and deterioration in rural infrastructure meant that most pregnant women in the countryside lost access to affordable maternal health services [12]. Furthermore, the economic crisis in the country largely contributed towards the impoverishment of many households particularly those living in rural areas which significantly reduced their odds of affording the maternal care services. Also, the intensification of the economic crisis meant that other health facilities ceased operating particularly in the rural areas where most of the average to poor wealth households reside [56]. The crisis period was also characterized by massive declines in donor funding to Zimbabwe which exerted pressure on the health sector as the amount of health financing dwindled. The closure of many health centers coupled with increased cost of maternal care services for the remaining facilities resulted in a lack of access to health facilities which were mostly accessible to the poor and thus contributed to the rich-poor gap in inequalities. As noted in N Alam, M Hajizadeh, A Dumont and P Fournier [4], affordability is an essential factor that can explain why the gap between the wealthy and the have-nots widened [4].

Our study is not without its shortcomings. One of the deficiencies of this study is that, the factors identified to influence maternal health care outcomes do not necessarily have a causal interpretation. We do not make an attempt to ascertain a causal effect of the socioeconomic factors on the two maternal health care outcomes considered. One can only interpret the reported coefficients as mere correlations or associations between the explanatory variables and maternal health care outcomes. Another shortcoming of our study is that, some of the data recorded by the ZDHS on maternal health care use are based on self-reports of the interviewed women. There is the possibility of recall bias associated with such responses which potentially influences our findings. Despite the highlighted shortcomings, this study makes a significant contribution to the literature in developing countries particularly sub-Saharan Africa.

## Conclusions

This study measured and explained inequalities in prenatal care, and professional delivery assistance in Zimbabwe. We found a pro-rich distribution of inequalities in professional delivery support and prenatal care over the 2005/06 and 2010/11 periods regardless of the place of residence. The observed pro-rich distribution in disparities in maternal health care was mostly explained by household wealth, education, religion, health insurance coverage, and access to information. The results of this study suggest the need for public health decision makers in Zimbabwe to concentrate on the most vulnerable segments of the population, especially those from low-wealth families and living in the countryside. Overall, the fact that maternal education and wealth are the main underlying drivers of inequalities in maternal health care in Zimbabwe suggests the need for a multi-sectoral approach to addressing these disparities.

## Abbreviations

DHS: Demographic and health survey
OECD: Organization for economic co-operation and development
PHC: Primary health care
SSA: Sub-Sahara Africa
ZDHS: Zimbabwe demographic and health survey
ZIMSTAT: Zimbabwe national statistics agency
